# Supplementary material for: A Modular Hepatitis E Virus Replicon System for Studies on the Role of ORF1-Encoded Polyprotein Domains
Source: Pathogens. 2022 Mar 15;11(3):355. doi: 10.3390/pathogens11030355 (PMC8948863; doi:10.3390/pathogens11030355)
Supplement: Supplementary file 1 [file pathogens-11-00355-s001.zip › pathogens-1605085-supplementary.pdf]

Table S1: List of replicon plasmids

Name	Length (nt)	description
**Unmodified replicons**		
pMK2-p6LucA26	9704	Full-length replicon p6LucA26 in plasmid backbone pMK2; derived from JQ679013 and p6-Luc [19]
pMK2-p6GAALucA26	9704	RdRp GDD->GAA mutant of full-length replicon p6LucA26 in plasmid backbone pMK2; derived from JQ679013
pMK2-47832mcLucA26	9697	Full-length replicon 47832mcLucA26 in plasmid backbone pMK2; derived from MN756606
pMK2-rab52LucA26	9573	Full-length replicon rab52LucA26 in plasmid backbone pMK2; derived from KY436898
pMK2-rab81LucA26	9500	Full-length replicon rab81LucA26 in plasmid backbone pMK2; derived from MT920909
**MYP chimeras**		
pMK2-p6(MYP.47832mc)LucA26	9703	Chimeric replicon p6(MYP.47832mc)LucA26 in plasmid backbone pMK2; p6LucA26 backbone with nt 1-2139 replaced by the homologous fragment (nt 1-2138) of 47832mcLucA26
pMK2-p6(MYP.rab52)LucA26	9705	Chimeric replicon p6(MYP.rab52)LucA26 in plasmid backbone pMK2; p6LucA26 backbone with nt 1-2139 replaced by the homologous fragment (nt 1-2140) of rab52LucA26
pMK2-p6(MYP.rab81)LucA26	9705	Chimeric replicon p6(MYP.rab81)LucA26 in plasmid backbone pMK2; p6LucA26 backbone with nt 1-2139 replaced by the homologous fragment (nt 1-2140) of rab81LucA26
**VXH chimeras**		
pMK2-p6(VXH.47832mc)LucA26	9704	Chimeric replicon p6(VXH.47832mc)LucA26 in plasmid backbone pMK2; p6LucA26 backbone with nt 2144-4059 replaced by the homologous fragment (nt 2143-4058) of 47832mcLucA26
pMK2-p6(VXH.rab52)LucA26	9575	Chimeric replicon p6(VXH.rab52)LucA26 in plasmid backbone pMK2; p6LucA26 backbone with nt 2144-4059 replaced by the homologous fragment (nt 2145-3931) of rab52LucA26
pMK2-p6(VXH.rab81)LucA26	9509	Chimeric replicon p6(VXH.rab81)LucA26 in plasmid backbone pMK2; p6LucA26 backbone with nt 2144-4059 replaced by the homologous fragment (nt 2145-3865) of rab81LucA26
**RJ chimeras**		
pMK2-p6(RJ.47832mc)LucA26	9704	Chimeric replicon p6(RJ.47832mc)LucA26 in plasmid backbone pMK2; p6LucA26 backbone with nt 4064-5347 replaced by the homologous fragment (nt 4063-5346) of 47832mcLucA26
pMK2-p6(RJ.rab52)LucA26	9704	Chimeric replicon p6(RJ.rab52)LucA26 in plasmid backbone pMK2; p6LucA26 backbone with nt 4064-5347 replaced by the homologous fragment (nt 3936-5219) of rab52LucA26
pMK2-p6(RJ.rab81)LucA26	9704	Chimeric replicon p6(RJ.rab81)LucA26 in plasmid backbone pMK2; p6LucA26 backbone with nt 4064-5347 replaced by the homologous fragment (nt 3870-5153) of rab81LucA26

Table S2: Sequences of cloning and sequencing primers.

Name	Sequence (5‘ -> 3‘)	Source	Used in
32c_3af	AGTCGAAGACATGGTCAACATCTGGTTTTTCTAG	this work	Cloning of VXH.47832mc
32c_3ar	CAGTGAAGACTTGACCATGTCCGGGTGTAC	this work	Cloning of MYP.47832mc
32c_4f	AGTCGAAGACTATGCCTCAGGAGCTTACG	this work	Cloning of RJ.47832mc
32c_4r	AGTCGAAGACGAGGCATATATAACAAGCCCTG	this work	Cloning of VXH.47832mc
32c_5r	AGTCGAAGACTCCCATGGGCAATGCACAA	this work	Cloning of RJ.47832mc
32c_6f	AGTCGAAGACCTGATGTTAGGATTCTAGTTCAAC	this work	Cloning of terA26.47832mc
52_3f	AGTCGAAGACTTGGTCCACGTCTGG	this work	Cloning of VXH.rab52
52_3r	AGTCCGTCTCGGACCAAGTTTTAGTATAAAGAGTC	this work	Cloning of MYP.rab52
52_4f	AGTCGAAGACTATGCCGCAGGAGCTTAC	this work	Cloning of RJ.rab52
52_4r	AGTCGAAGACTCGGCATATACAGGAGACCC	this work	Cloning of VXH.rab52
52_5r	AGTCGAAGACTCCCATGGGCAATGCAG	this work	Cloning of RJ.rab52
52_6f	AGTCGAAGACTCGATGTCAGGATCCTTGTTC	this work	Cloning of ter.rab52
52fw_bHel	CCAGATTAGTGCCTACCACC	this work	sequencing
52fw_Hel	TGCTGGCTGTACCGTTAACC	this work	sequencing
52fw_HVR	GTCCAGCTGCCACCTTCTG	this work	sequencing
52fw_MT	ACCTACCACACTACCTCTTAC	this work	sequencing
52fw_ORF2	ATACCTCATGACATCGATCTTG	this work	sequencing
52fw_PCP	GTGAAGAGTGTGACCAAGAAG	this work	sequencing
52fw_Rdrp	GTTATATCATCTGGTCCGGTC	this work	sequencing
52rv_ORF2	GTCTGATCGTACTCAGCAGC	this work	sequencing
52s_3f	ACTTGGTCCACGTCTGG	this work	sequencing
52s_4f	TATGCCGCAGGAGCTTAC	this work	sequencing
52s_4r	CGGCATATACAGGAGACCC	this work	sequencing
52s_5r	TCCCATGGGCAATGCAG	this work	sequencing
81.3'250	ACTGTTGACCACCCTGCACG	this work	sequencing
81_3f2	AGTCGAAGACTTGGTCAACATCTGGTT	this work	Cloning of VXH.rab81
81_3r	AGTCGAAGACTTGACCAAGTCCGAGTATACAAG	this work	Cloning of MYP.rab81
81_4f	AGTCCGTCTCATGCCGCAGGAGCTTAC	this work	Cloning of RJ.rab81
81_4r	AGTCGAAGACGCGGCATGTAAAGCAACC	this work	Cloning of VXH.rab81
81_5r	AGTCCGTCTCCCCATGGGCAATGCACAG	this work	Sequencing; Cloning of RJ.rab81

Table S2 (continued)

Name	Sequence (5‘ -> 3‘)	Source	Used in
81_6f	AGTCGAAGACCTGATGTCAGGATTCTGGTC	this work	Cloning of ter.rab81
AmpRp_rv	CTGAGAAGACCATACTCTTCCTTTTTCAATATTATTGAAGC	this work	Sequencing Cloning of pMK2
BbsI_T(26)CC	GAAGACGCTTTTTTTTTTTTTTTTTTTTTTTTTTCC	this work	Cloning of terA26.rab52 terA26.rab81 terA26.47832mc
BbsI_T7p	GAAGACTTCTGCTAATACGACTCACTATA	this work	Cloning of MYP.rab81 MYP.47832mc
Cloning of Analysis Forward Primer	ACCTGCCAACCAAAGCGAGAAC	NEB	sequencing
Cloning of Analysis Reverse Primer	TCAGGGTTATTGTCTCATGAGCG	NEB	sequencing
Esp3I_T(26)CC	CGTCTCGTTTTTTTTTTTTTTTTTTTTTTTTTTCC	this work	Cloning of GLuc_terA26.p6
Esp3I_T7p	CGTCTCGCTGCTAATACGACTCACTATA	this work	Cloning of MYP.p6 MYP.rab52
F1610	CTTGAGGCCCTTTATAGTGC	[29]	sequencing
F2991c	CTATTCAACAGGGTGACGTTG	this work	sequencing
F5086	GGACCTTACAAATTCTATTATACAG	[29]	sequencing
F6485	CGACAATCAACATGAACAGGAC	[29]	sequencing
F705	GTGCAGGGTATAACCATGATG	[29]	sequencing
GLuc_fw_Kp6_O2	CTGACGTCTCCATGGGATCACCATGGGAGTCAAAGTTCTGTTTG	this work	Cloning of GLuc-terA26.p6
K2_rv	CCTTGATTACGGTAGTGGAG	this work	sequencing
KanR_fw	CTGAGAAGACAGAGTATGATTGAACAAGATGGATTGC	this work	Cloning of pMK2
KanR_rv	CTGAGAAGACAGAGTCAGAAGAACTCGTCAAGAAG	this work	Cloning of pMK2
Kp6/GAA_fw	CTGACGTCTCGCAGCTTCGGTGGTCCTCTGTAGCG	this work	Cloning of RJ_GAA.p6
Kp6/GAA_rv	CTGACGTCTCAGCTGCACCCTTAAAGGCAGCAACAC	this work	Cloning of RJ_GAA.p6
Kp6_3af	CAGTCGTCTCTGGTCAACATCTGGCTTTTC	this work	Cloning of VXH.p6
Kp6_3ar	CAGTCGTCTCTGACCAGGTCCGGGTATAC	this work	Cloning of MYP.p6
Kp6_4f	CAGTCGTCTCATGCCACAAGAGCTTACCGTG	this work	Cloning of RJ.p6
Kp6_4r	CAGTCGTCTCTGGCATGTAAAGCAGGCCCTG	this work	Cloning of MYP.p6
Kp6_5r	CAGTCGTCTCCCCATGGGCGATGCAACAAAC	this work	Cloning of RJ.p6

Table S2 (continued)

Name	Sequence (5‘ -> 3‘)	Source	Used in
Ori_fw	CTGA<u>GAAGAC</u>GAGACTGTCAGACCAAGTTTACTC	this work	Cloning of pMK2
p15A_seq_rv	GAGTCAGTGAGCGAGGAAG	this work	sequencing
p6_F1750	GGACGACGGTGGTTGA	this work	sequencing
p6_F2700	AGACTGTGATTGGCTGG	this work	sequencing
p6_F3250	GATGTTGATGTGGTGGTTG	this work	sequencing
p6_F4000	TCCTGCCAGATTAGTGC	this work	sequencing
p6_F4900	GGTCCTCTGTAGCGAC	this work	sequencing
p6_F600	TTGTATGCYGCACTACATCT	this work	sequencing
p6_F6750	CCAATGGCGAGCCGAC	this work	sequencing
p6_R1900	ATGTAGCAGTGCAATCCAG	this work	sequencing
p6_R2900	TGGTATATGCCCGAGCC	this work	sequencing
p6_R4300	CTCCACCATGGCCTCAA	this work	sequencing
p6_R5300	CCAATAAGGTTATGTACCAG	this work	sequencing
p6_R6100	ATGRAGGCGCTCACTAG	this work	sequencing
p6_R6800	TCGTACTCAGCGGCAGT	this work	sequencing
pACYC_Seq	AATAGGCGTATCACGAGGC	this work	sequencing
pMK2_fw	CGGGATCCGAAGACTGAAAATTTAAATACGAAAGGGCCTCGTGATAC	this work	Cloning of pMK2
pMK2_rv	CGGGATCCGAAGACAGGCAGTCCTAGCGGAGTGTATACTGG	this work	Cloning of pMK2
Primer_2F	GACTACTATTATTGCTACGGC	[29]	sequencing
Primer_3F	GAGATGATTCAATGGTCTTATG	[29]	sequencing
Primer_4R	CACATAAAATGTTTTAGAATGC	[29]	sequencing
R2429	CACAATCCGACTCGAATAAGG	[29]	sequencing
R3157	CAGCAAATGCGGTGGTAATGAC	[29]	sequencing
R5625	CATACAAGACAAGATTAGTGCC	[29]	sequencing

Underlined nucleotides represent the recognition sites of the restriction endonucleases used for cloning.

Table S3: List of Cloning of intermediates

**Name**	**Length (nt)**	**description**
**<u>MYP Fragments</u>**		
pMiniT2.0-T7pMYP.47832mc	4771	subgenomic fragment MYP.47832mc with T7 promoter in pMiniT2.0
pMiniT2.0-T7pMYP.rab52	4771	subgenomic fragment MYP.rab52 with T7 promoter in pMiniT2.0
pMiniT2.0-T7pMYP.rab81	4773	subgenomic fragment MYP.rab81 with T7 promoter in pMiniT2.0
pMiniT2.0-T7pMYP.p6	4770	subgenomic fragment MYP.p6 with T7 promoter in pMiniT2.0
**<u>VXH Fragments</u>**		
pMiniT2.0-VXH.47832mc	4536	subgenomic fragment VXH.47832mc in pMiniT2.0
pMiniT2.0-VXH.rab52	4407	subgenomic fragment VXH.rab52 in pMiniT2.0
pMiniT2.0-VXH.rab81	4341	subgenomic fragment VXH.rab81 in pMiniT2.0
pMiniT2.0-VXH.p6	4534	subgenomic fragment VXH.p6 in pMiniT2.0
**<u>RJ Fragments</u>**		
pMiniT2.0-RJ.47832mc	3904	subgenomic fragment RJ.47832mc in pMiniT2.0
pMiniT2.0-RJ.rab52	3904	subgenomic fragment RJ.rab52 in pMiniT2.0
pMiniT2.0-RJ.rab81	3902	subgenomic fragment RJ.rab81 in pMiniT2.0
pMiniT2.0-RJ.p6	3902	subgenomic fragment RJ.p6 in pMiniT2.0
pMiniT2.0-RJ_GAA.p6	3902	subgenomic fragment RJ_GAA.p6 in pMiniT2.0; contains RdRp GDD->GAA codon exchanges
**<u>Gaussia Luciferase reporter and terminal fragment of p6LucA26</u>**		
pMiniT2.0-insGLuc-terA26.p6	4881	subgenomic fragment insGLuc-terA26.p6 in pMiniT2.0
**<u>3’ Terminal fragments</u>** of 47832mc, rab52, rab81, for combination with *Gaussia* luciferase reporter fragments		
pMiniT2.0-terA26.47832mc	3995	subgenomic fragment terA26.47832mc in pMiniT2.0
pMiniT2.0-terA26.rab52	3998	subgenomic fragment terA26.rab52mc in pMiniT2.0
pMiniT2.0-terA26.rab81	3991	subgenomic fragment terA26.rab81 in pMiniT2.0
**<u>Gaussia Luciferase reporter fragments</u>** for combination with corresponding _terA26 of 47832mc, rab52, rab81		
32cLuc_GL1 in pTwist Amp High Copy	3121	contains 11nt upstream of the ORF2 start codon, a *Gaussia* luciferase reporter inserted at the ORF2 start codon (deleting 377nt of the HEV genome), and 317nt of the 47832mc genome
52Luc_GL1 in pTwist Amp High Copy	3121	contains 11nt upstream of the ORF2 start codon, a *Gaussia* luciferase reporter inserted at the ORF2 start codon (deleting 377nt of the HEV genome), and 317nt of the rab52 genome
81Luc_GL1 in pTwist Amp High Copy	3121	contains 11nt upstream of the ORF2 start codon, a *Gaussia* luciferase reporter inserted at the ORF2 start codon (deleting 380nt of the HEV genome), and 317nt of the rab81 genome
**<u>Cloning of backbone plasmid for replicon constructs</u>**		
pMK2	2101	Cloning of vector pMK2; Kanamycin resistance gene, pBR322-derived origin of replication

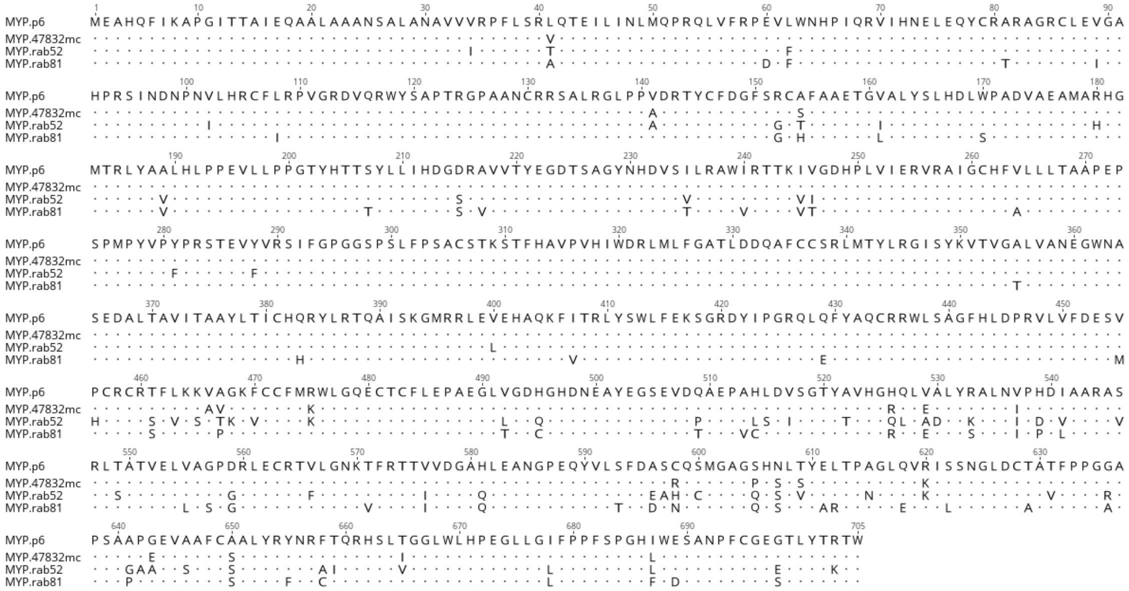

Figure S1: Multiple amino acid sequence alignment of the ORF1-MYP-encoded protein segment from the model strains p6, 47832mc, rab52, rab81. The sequences were alignmed with MUSCLE in Geneious. Dots indicate residues which are identical to the corresponding position in p6. Numbers indicate the amino acid position in p6.

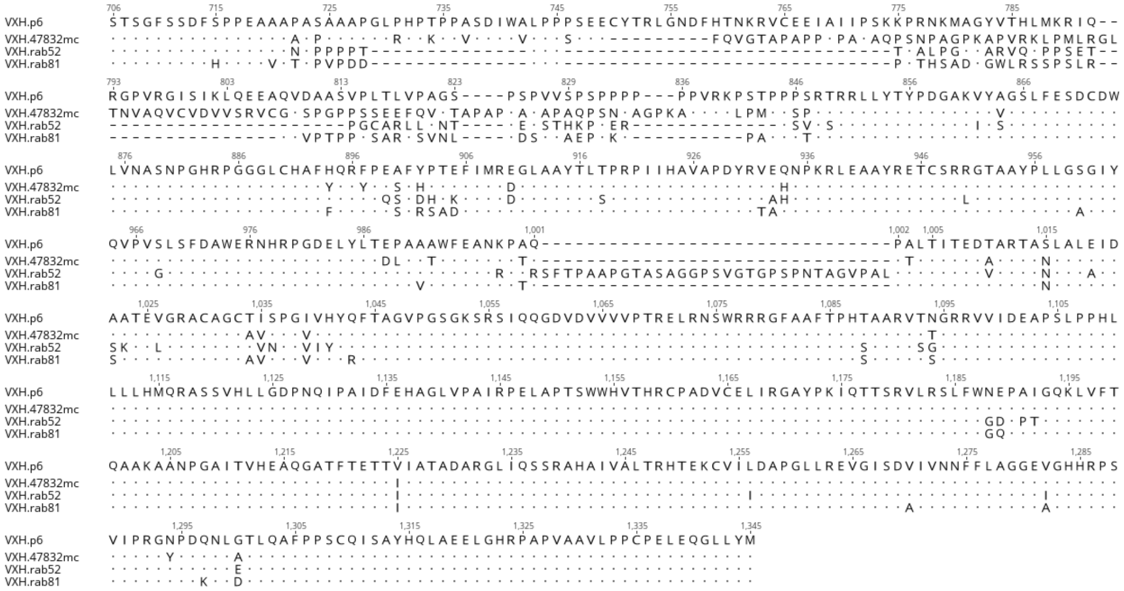

Figure S2: Multiple amino acid sequence alignment of the ORF1-VXH-encoded protein segment from the model strains p6, 47832mc, rab52, rab81. The sequences were alignmed with MUSCLE in Geneious. Dots indicate residues which are identical to the corresponding position in p6. Numbers indicate the amino acid position in p6.

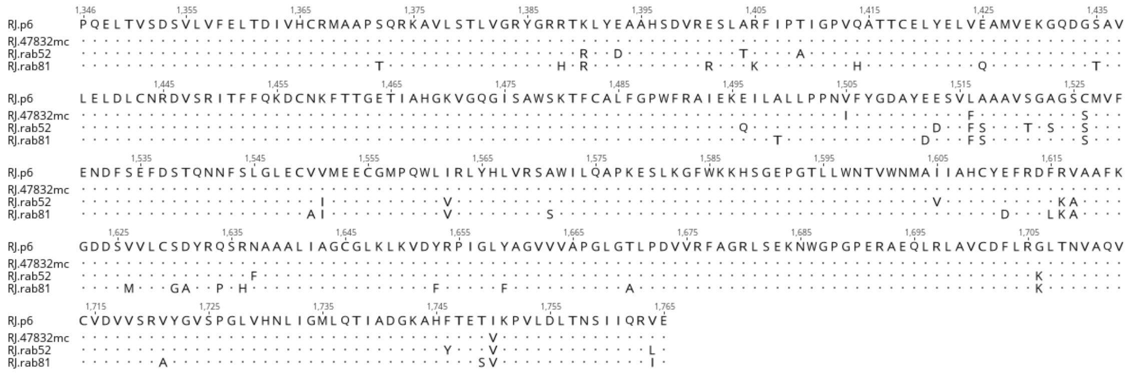

Figure S3: Multiple amino acid sequence alignment of the ORF1-RJ-encoded protein segment from the model strains p6, 47832mc, rab52, rab81. The sequences were alignmed with MUSCLE in Geneious. Dots indicate residues which are identical to the corresponding position in p6. Numbers indicate the amino acid position in p6 [19,29].

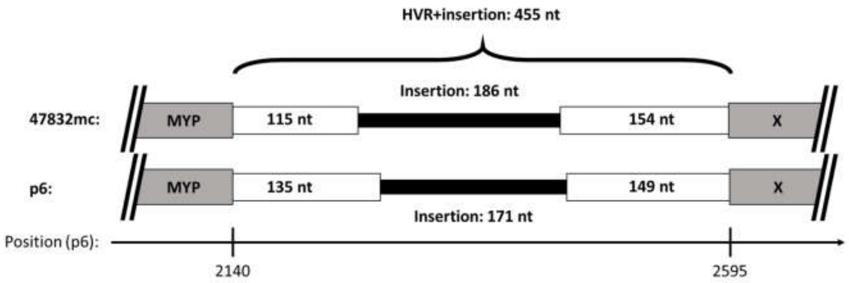

Figure S4: Comparison of the HVR insertions of p6 and 47832mc. While the insertion of p6 is shorter than the insertion within the HVR of 47832mc by 15 nucleotides, remainder of the p6 HVR is longer by the same amount. As a consequence, the total lengths of both HVRs are identical [19,29].
